# The neural differences of arithmetic verification performance depend on math skill: Evidence from event‐related potential

**DOI:** 10.1002/npr2.12158

**Published:** 2021-01-18

**Authors:** Shiva Taghizadeh, Touraj Hashemi, Ali Jahan, Mohammad Ali Nazari

**Affiliations:** ^1^ Division Cognitive Neuroscience Faculty of Education and Psychology University of Tabriz Tabriz Iran; ^2^ Department of Psychology Faculty of Education and Psychology University of Tabriz Tabriz Iran; ^3^ Brain and Cognition Lab Tabriz University of Medical Sciences Tabriz Iran

**Keywords:** arithmetic verification, ERP, mathematic education, mental arithmetic, numerical cognition

## Abstract

**Aim:**

Math skill is a basic need for an individual, as a career prospect. However, little is known about early brain processes of arithmetic between individuals with different math skill. Therefore, we questioned the modulation of the amplitude of an early negative component by math skill level in an arithmetic verification paradigm using event‐related potential (ERP).

**Methods:**

Thirty‐six right‐handed participants were assigned in two groups of high‐ and low‐performing students. Their electroencephalogram was recorded while they completed an arithmetic verification task. Simple arithmetic operands were made by random digits from 1 to 9. Addition and subtraction operations were equally used in correct and incorrect responses. The accuracy scores, reaction times, and peak amplitude of the negativity in 200‐400 ms time window were analyzed.

**Results:**

The high‐performing group showed significantly higher response speeds, and they were more accurate than the low‐performing group. The group × region interaction effect was significant. The high‐performing group showed a significantly greater negativity, particularly in parietal region, while the low‐performing group showed a significantly deeper negativity in frontal and prefrontal region. In the low‐performing group, there were significant peak amplitude differences between the anterior and posterior areas. However, such differences were not detected in the high‐performing group.

**Conclusion:**

Students with different mathematical performance showed distinct patterns in early processing of arithmetic verification, as reflected by differences in negativity at 200‐400 ms at anterior and posterior. This suggests that ERPs could be used to differentiate math mastery at neural level which is beneficial in educational and clinical contexts.

## Abstract 2

Students with different mathematical performance showed distinct patterns in early processing of arithmetic verification, as reflected by differences in negativity at 200‐400 nonbreakingspace

ms at anterior and posterior.

## Graphical abstract

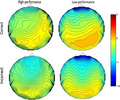

## 1 INTRODUCTION

Specialized neural networks in the brain equip us with arithmetic and mathematical abilities. Renovations are made in education in line with the findings on brain processes. An increasing number of studies have examined the mental arithmetic processes with neuroimaging or electrophysiological techniques. These studies help us to understand learning‐related cognitive processes, structure educational environments and provide materials to increase the mathematical competencies.^1^ For achieving academic, social, and economic success, a competent performance in mathematics is essential that could remarkably affect the future and well‐being of the individuals.^2^

Proficiency in integrating fundamental numerical concepts is essential for developing a complicated skill, such as mathematics.^3^, ^4^ Hence, identifying the leading and predictive skills associated with success in mathematics is critical for both theoretical and practical reasons. Previous studies have pinpointed a range of basic numerical competencies, which enable scientists to evaluate individuals in terms of different mathematical skills. For instance, in some studies, it has been argued that the number comparison is essential for numerical magnitude processing.^5^, ^6^, ^7^ Neuroimaging studies have found relationships between the brain activation while performing a number comparison task and mathematical achievement. However, this association is still a subject of controversy, especially with tools with high temporal resolution such as event‐related potential (ERP).

ERP researches on number cognition and mathematics were mostly done with two kinds of tasks namely production tasks and verification tasks. In the first task, participants have to calculate and produce the accurate response of calculation problems. In the verification task, the participants were asked to verify if the presented response was correct or incorrect. During completing different arithmetic tasks, several components of ERPs have been observed. For instance, Szucs and Csepe (2011) reported a mismatch negativity (MMN) in the 240‐300 ms time window, which they called arithmetic mismatch negativity (AMN), using the number matching task. This negative‐going potential while employing the number matching and verification tasks was reportedly more negative in false mathematical equations than correct ones. This component has been given different labels, such as N270, N2b, N400, and N300.^8^, ^9^, ^10^

Wang Y. et al (2000) recorded 15 participant's EEGs with an arithmetic task, which involved two conditions. They reported a negative potential in condition 2 (false answer to the anticipated mental computation) with peak latency of about 270 ms (N270). They concluded that N270 indicated the endogenous mental conflict processing in human brain. This process is a general and automatic cognitive ability.^11^ Later, the same team conducted another experiment to explore if this component was also evoked in a number discrimination task.^9^ In this setup, number pairs were presented and 14 right‐handed participants decided whether a number pair was the same (match condition) or not (mismatch condition). They have seen a negativity (N270) in mismatch conditions, particularly at the central and occipital areas. They pointed this neurophysiological event as a reflection of the brain number mismatch processing.

The ERPs evoked by incorrect results may relate to the violated expectations in the activated nodes of an arithmetic storage network.^12^ Studies have shown that MMN in verification task could reveal strategic expectation of participant to the ratio of correct/incorrect responses. Avancini (2015) reported a negative component (330‐400 ms), as a strategic expectation effect, in raw ERPs in left frontal versus right parietal. They demonstrated that detection of arithmetic correctness temporally overlaps with detection of the violation of strategic expectations.^13^

Individual differences in math skill can be reflected in ERP patterns underlying arithmetic processing as well. However, less is known about the impacts of math skill on the early processes of calculation. It is interesting to know whether mastery in a more abstract processing like mental arithmetic could be tracked back to earlier automatic general process. In this regard, the main aim of this study is to investigate whether this negativity (AMN) evoked by arithmetic verification task with the same ratio of correct/incorrect responses could differentiate high/low performers in terms of math skills. If there is a difference between two groups, it may reflect the math skills‐related neural activity, which could be considered as an arithmetic brain potential in early stages of processing.

## 2 METHODS

### 2.1 Participants

Forty‐seven right‐handed students (28 females and 19 males) within the age range of 19‐33 years (mean age 22.85 ± 4.37) participated in this study. All received more than 12 years of education. They were divided into two groups based on their performance in the calculation task: 22 high‐performing (HP) and 25 low‐performing (LP) groups. Data from 11 participants were discarded due to high artifact in EEG. Data of thirty‐six (19 females, 17 males, mean age 23.5 ± 4.42) including 18 HP individuals and 18 LP individuals were analyzed. All groups had normal or corrected‐to‐normal visions and were Persian speakers with no reported history of learning difficulties, no psychiatric/neurological disorders/diseases nor use of medications that might affect neural function (eg, medication for depression or seizure). All participants signed the written informed consent.

### 2.2 Stimuli and task

**FIGURE 1:**
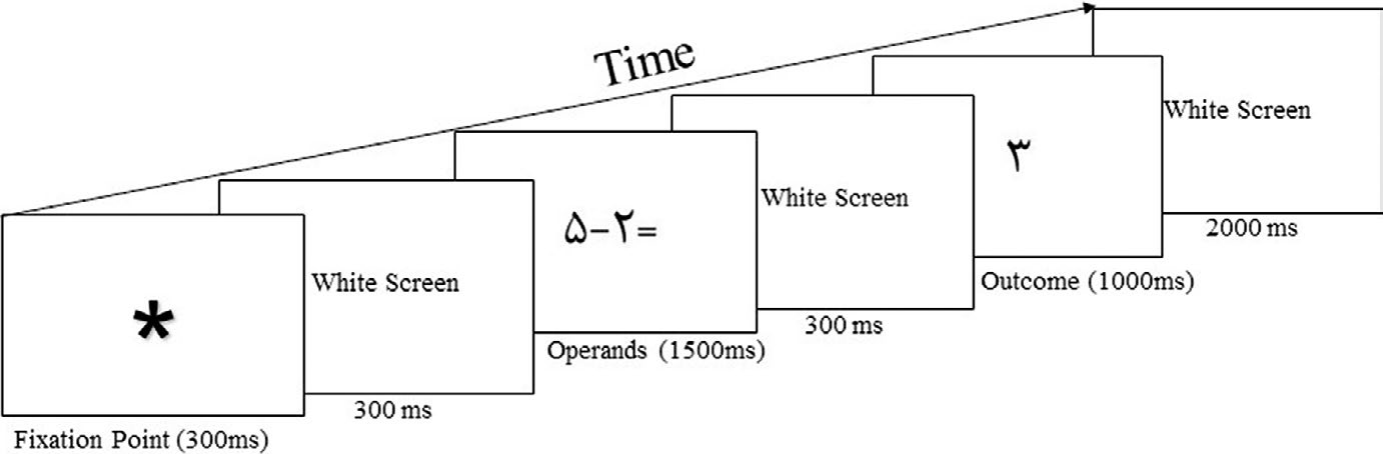
Presentation order of the stimulus in the experiment. Stimuli numbers are in eastern Arabic numerals and the shown calculation pattern is (5 − 2 = 3)

Visual stimuli were shown on a monitor located 70 cm in front of the participants. A stimulus system eevoke™ (synchronized with EEG amplifier) was employed for controlling the stimulus presentation during the acquisition of ERP signals. The black stimuli were surrounded with a white background. One hundred and twenty trials were presented to each subject. The schematic graph for the stimulus presentation is shown in Figure 1.

Stimuli in each trial produced a calculation pattern (like 5 − 2 = 3). Each trial began with fixation point (a star) at the center of the screen that lasted 300 ms Three hundred milliseconds after the offset, the second stimuli (arithmetic operation, eg, “5 − 2=”) appeared on the screen for 1500 ms; after 300 ms interstimulus interval, the last stimulus (a number as the answer for the operation eg, “3”) was presented for 1000 ms Addition and subtraction were equally employed. Digits from 1 to 9 had the same opportunity to be the outcome, and the numerical distance between correct answer and the stimulus shown as outcomes was either one or two. The fixation point of the second trial was presented 2 seconds after the offset of the last stimulus of the preceded trial. Trials were in two different conditions: condition 1 (correct answer; like 4 − 3 = 1), and condition 2 (incorrect answer; like 2 + 3 = 4). Each of the conditions involved sixty trials so the ratio of correct/incorrect responses was controlled. Experimental session lasted for about 50 minutes.

Participants were told they would see a series of mathematical problems and they should make a judgment about the correctness of the outcome displayed at the end of each problem. They were asked to right click (with index finger) if the answer was correct and left click if it was incorrect as soon as possible after the onset of the last stimulus. The left and right button pressing was counterbalanced. The session started with a short practice block that did not include any of the experimental problems. For each subject and condition, the mathematical problems were presented randomly. Following the presentation of stimulus, subjects began to calculate and match their arithmetic results to the given answer. Numbers were in Eastern Arabic numeral form.

### 2.3 EEG recording and signal pre‐processing

The EEG was recorded from 64 Ag/AgCl electrodes arranged according to the 10‐10 system inserted in a WaveGuard EEG Cap (ANT Company, Netherlands). A linked mastoid reference was used with the ground electrode located on AFz; EEG cancelation was minimized by this montage.^14^ Impedance was kept below 10 kΩ. The EEG was recorded and digitized by an ANT 64‐channel amplifier (ANT Company) with a sampling rate of 250 Hz. Participants were instructed to avoid body or eye movements at the time of recording as much as possible. The raw EEG signals were first high‐pass filtered above 0.5 Hz and low‐pass filtered below 40 Hz, to eliminate line noise and other artifacts by a windowed FIR sync filter. The EEG recordings were then visually examined for any artifacts, and the epochs with artifacts were rejected from further processing. The eye blinks and muscle tensions were identified and removed via the Independent Component Analysis (ICA). The EEG data were processed using EEGLAB functions toolbox.^15^

### 2.4 Data analysis

#### 2.4.1 Behavioral data

Reaction time (RT) and accuracy (ACC = correct answers/all × 100) were calculated for each participant and experimental condition. Via Repeated‐measures ANOVA, the mean data between the two conditions in each group were compared.

#### 2.4.2 ERP data

Epochs were extracted from −200 ms to 1000 ms relative to the second stimulus presentation. From the de‐noised set of raw data, ERPs were extracted for each subject by averaging single trials separately for electrodes and each experimental condition. For this purpose, the correct responded trials were used. ERPs were measured as peak amplitude in the 200‐400 ms latency windows following the onset of the outcome. For data reduction purpose, data from 64 channels were converted into eight regions (see Table 1). A repeated‐measures ANOVA was implemented to reveal the difference between groups in terms of ERP amplitude in correct and incorrect conditions. The group (low‐/high performing) was used as between‐subject factor and condition (correct/incorrect), region (prefrontal, frontal, frontocentral, central, centroparietal, parietal, parieto‐occipital, occipital), electrode laterality (left, central, right) were used as within‐subject factors. As the sphericity assumption was violated, Greenhouse‐Geisser F was reported. Any interaction of factors with group was assumed to answer the study questions. Further post hoc analysis was done whenever needed.

**TABLE 1:** The order of channels, which used for data analyses of regions

Region	Laterality	Channels which averaged for this region			
FP	Midline	FPz			
	Right	FP2	AF4	AF8	
	Left	FP1	AF3	AF7	
F	Midline	Fz			
	Right	F2	F4	F6	F8
	Left	F1	F3	F5	F7
FC	Midline	FCz			
	Right	FC2	FC4	FC6	FT8
	Left	FC1	FC3	FC5	FT7
C	Midline	Cz			
	Right	C2	C4	C6	T8
	Left	C1	C3	C5	T7
CP	Midline	CPz			
	Right	CP2	CP4	CP6	TP8
	Left	CP1	CP3	CP5	TP7
P	Midline	Pz			
	Right	P2	P4	P6	P8
	Left	P1	P3	P5	P7
PO	Midline	POz			
	Right	PO4	PO6	PO8	
	Left	PO3	PO5	PO7	
O	Midline	Oz			
	Right	O2			
	Left	O1			

Note

All 64 channels were used.

## 3 RESULTS

### 3.1 Behavioral results

**FIGURE 2:**
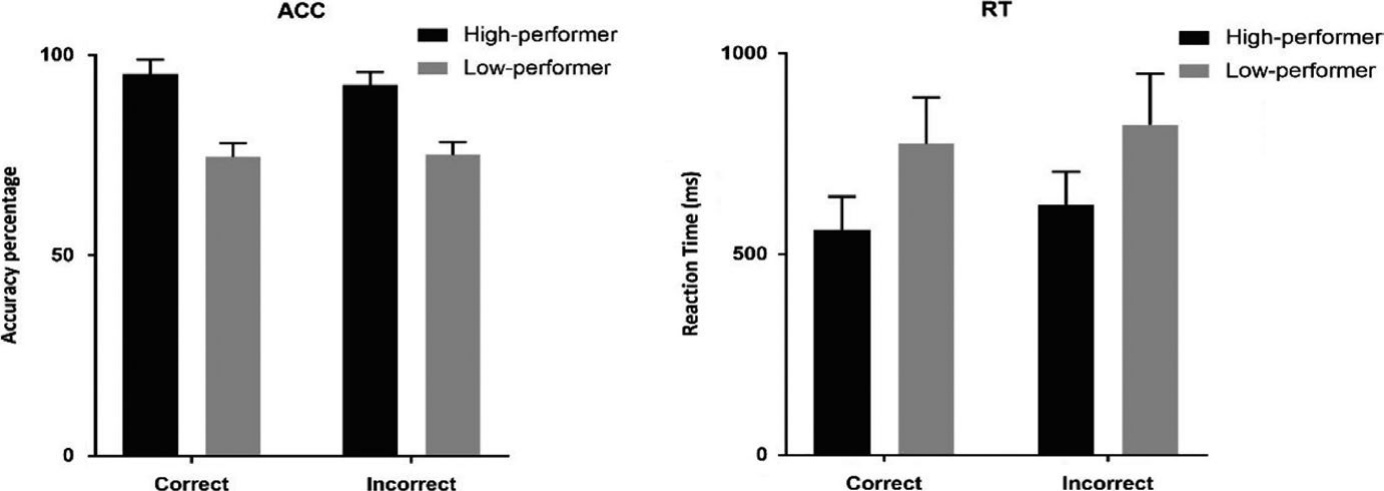
Mean Accuracy and Reaction times of high‐performing and low‐performing groups in correct and incorrect conditions. In both conditions, high‐performing group has significantly more accurate and short reaction time than low‐performing group

RTs in the low‐performing group for correct condition ranged from 596.50 to 967.94 ms (mean = 775 ± 116.41 ms) and for the incorrect condition, ranged from 608.70 to 1038.08 ms (mean = 822.91 ± 126.32 ms). RTs for high‐performing group ranged from 433.32 to 696.29 ms (mean = 563.37 ± 84.41 ms) and ranged from 424 to 743.08 ms, (mean = 628.40 ± 81.99 ms) for the correct and incorrect conditions, respectively. Repeated‐measures ANOVA revealed that the main effect of condition was significant [*F*(1, 36) = 27.127, *P* ≤ .001] in both groups; RTs were shorter to correct condition in comparison with incorrect condition. HP group significantly showed higher response speed compared to LPs (Figure 2 right panel).

HP group had a greater ACC value for correct condition (95.35%) than the LP group (74.75%) (*P* ≤ .001), as well as for incorrect condition, HPs (mean = 92.52%), LPs (mean = 75.16%) *P* ≤ .001 (Figure 2 left panel).

### 3.2 ERP results

**FIGURE 3:**
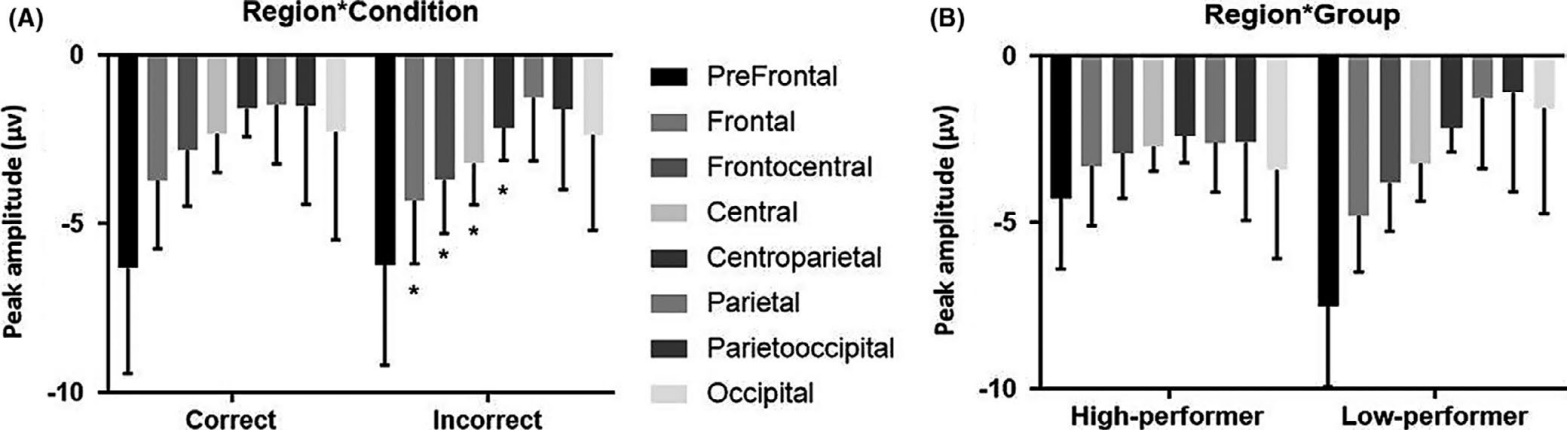
A, The interaction effect between region and condition. B, The interaction effect between region and group; average values for all electrodes are shown

**FIGURE 4:**
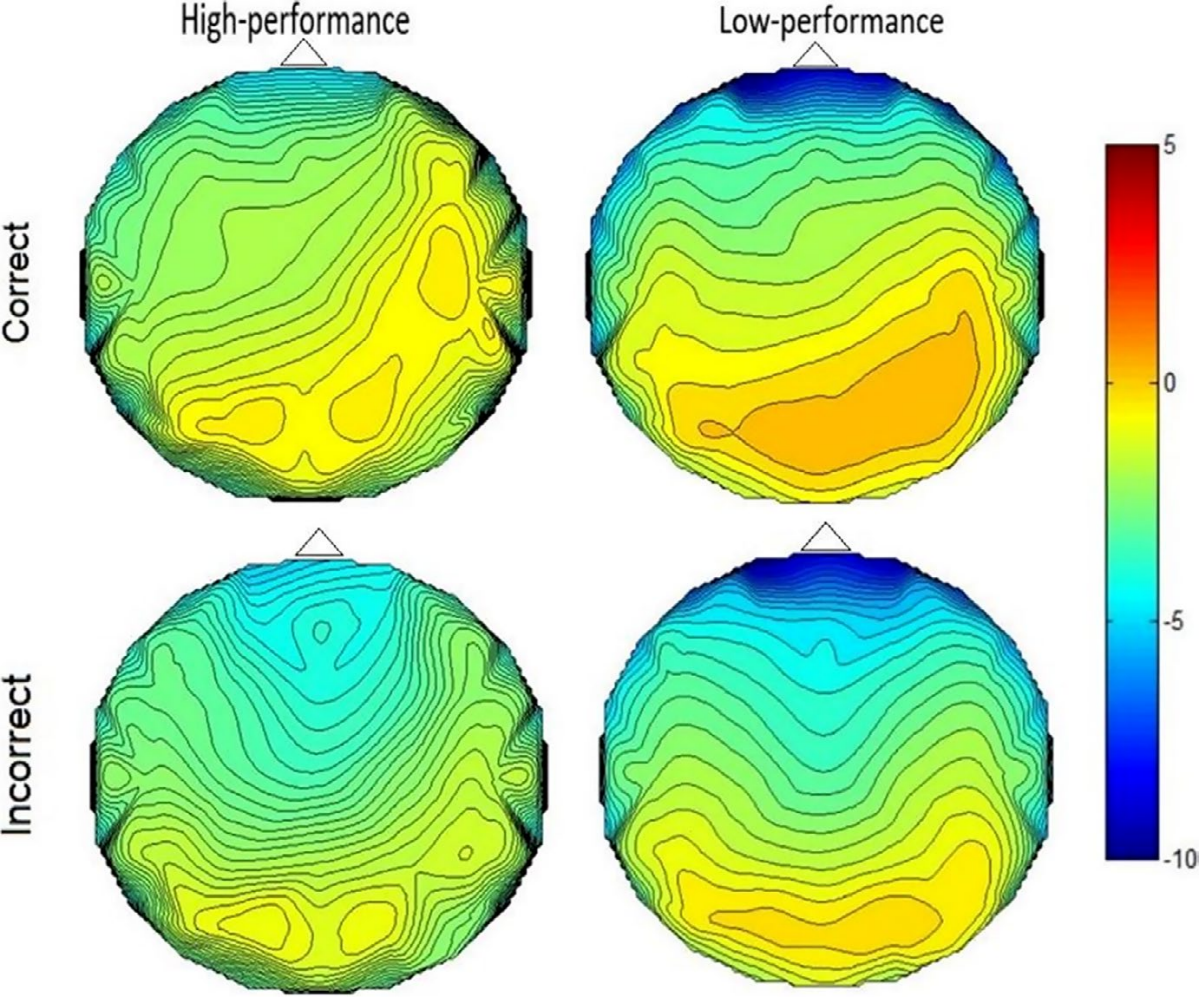
Scalp voltage (μV) topography according to groups and conditions, for the HP group (left column) and the LP group (right column) in the 200‐400 ms window for correct condition (upper panel) and incorrect condition (lower panel)

**FIGURE 5:**
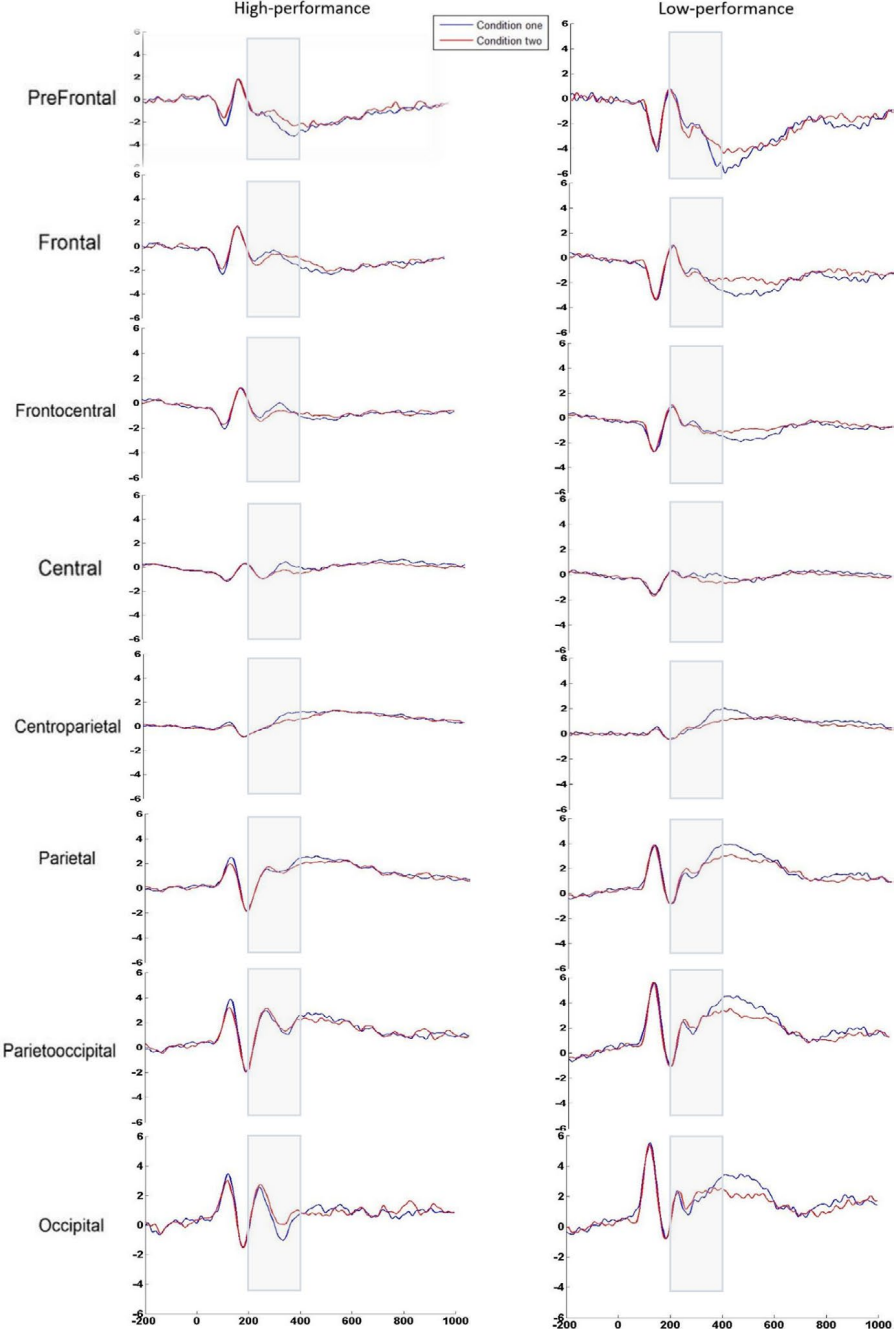
Negative components in the 200‐400 (ms) time window according to regions for both groups. Averaged waveforms in eight regions elicited by both correct (one) and incorrect (two) conditions (the voltage range for raw ERPs is ±6 μV)

The repeated‐measures ANOVA conducted on peak amplitude of the 200‐400 ms window showed statistically significant interactions for region × group [*F*(1.6, 58.8) = 8.06, *P* ≤ .001], for condition × laterality, [*F*(1.86, 66.9) = 12.07, *P* ≤ .001], and for condition × region [*F*(2. 64, 95.26) = 3.42, *P* ≤ .001]. Also, main effect of condition [*F*(1, 36) = 17.87, *P* ≤ .001], region [*F*(6.32, 58.89) = 26.77, *P* ≤ .001], and laterality [*F*(1.83, 66.03) = 4.60, *P* ≤ .001] was significant. As shown in Figure 3B, the negativity was found in low‐performing group significantly deeper at frontal and prefrontal than posterior regions [*F*(1.636, 58.892) = 8.068, *P* ≤ .001], with a reduction in posterior peak compared with the frontal region. In addition, it was more negative within condition incorrect than correct one. In contrast, in HP group across sites, the peak amplitude was not different from anterior to posterior sites (Figure 3B). Post hoc comparisons were conducted on the eight regions, separately. Disregarding group and laterality, peak amplitude at frontal, frontocentral, central and centroparietal regions differ significantly in two conditions (*t*(37) = 2.87; *t*(37) = 4.8; *t*(37) = 5.3; *t*(37) = 3.561025; *P* ≤ .001) (see Figure 3A). The condition effect was statistically significant for two levels of laterality (right side and midline) *t*(37) = 3.204; *t*(37) = 6.487 respectively; all *P*‐values ≤ .001, and the peak amplitude of this negativity in both conditions did not differ on the left side. Across right side to midline, the negativity was deeper in incorrect condition than correct condition (Figure 4). Waveform ERP is plotted in Figure 5 according to regions for both groups.

## 4 DISCUSSION

This study aimed to examine differences in an early negative potential of high/low‐skilled students in terms of arithmetic solution verification task. Hence, we questioned the modulation of the negative amplitude in 200‐400 ms window by skill level between low/high‐performing groups. In this study, ERP, as a neurocognitive method in neuroscience, in the framework of neuroeducational point in mathematics was applied.

In line with previous studies, HP performance was more accurate and faster compared to LP performance; this validates the former reports indicating a correlation between individual distinction in the arithmetic skills, with differences in their performance in terms of numerical processing tasks, such as verification or number comparison and weak performance associated with low mathematic skills.^6^, ^16^, ^17^ Similarly, some studies have shown that individual differences in the specification of the incongruity were pertinent to the math performance.^18^, ^19^, ^20^

In this study, the negative component was extracted by incongruent mental calculation response and subjects were required to perform calculation following the stimulus presentation step by step, and keep the calculation results in their mind waiting for the appearance of the answer. They simply matched the two numbers with little possibility relating them. In this study, the difference in negative peak amplitude between two groups at frontal, prefrontal, and parietal regions was revealed. A recent quantitative meta‐analytic study concluded that frontal and parietal regions could be the neural base of a general magnitude processing system for numerical magnitude processing.^21^ In the current study, deeper negativity of frontal and prefrontal regions was seen in LP group, which in turn could be associated with an underdeveloped numerical processing in this group. However, participants with more negativity in parietal areas showed better performance in terms of arithmetical task. This finding agrees with behavioral studies showing slower development of number‐related competencies in LP group and children with learning disability and those with working memory deficits.^4^

A meta‐analyze study, reviewing functional imaging researches on number processing, suggested that the regions overlapped during calculation and number processes, but prefrontal cortices are notably more involved in the calculation rather than number task. This might suggests greater cognitive resources engagements, for instance working memory, during calculation tasks.^22^ Overall, the EEG analysis tools have limited spatial resolution; therefore, this interpretation should be approached cautiously.

In contrast, we observed that individuals with more negative potential peak at parietal region probably process numbers automatically and show higher performance in task and have enhanced math skills. This assumption seems to agree with the findings of González (2018) who analyzed EEG coherence in children with different mathematical achievements during a symbolic magnitude comparison task and declared that the beta coherence was focalized at parietal regions in children with superior arithmetic skills. They concluded this as a reflection of the numerical processing that might suggest a higher grade of automation or system specialization in these children.^18^

Previous ERP studies showed slow waves differ by arithmetic difficulty between individuals with different math ability. For instance, Núñez‐Peña and colleagues (2011) found a slow positive wave at centroparietal sites. They indicated that high‐skilled versus low‐skilled participants show different positive wave denoting different strategies to get the calculation result. High‐skilled individuals retrieve the result from memory for small and medium problem sizes and calculate for large problem sizes. However, low‐skill individuals rely on calculation strategy for medium‐ and large‐size problems. It is concluded that they retrieve the answer for small sizes from memory.^23^

Systematically, three brain regions are involved in calculation: parietal, precentral, and prefrontal; the numerical distance effect for arithmetic solution verification tasks is found at an extensive distribution, including frontal, central, and parietal electrodes.^3^

In accordance with these findings, one MRI study with math gifted and normal control adolescents revealed that the math experts showed a greater surface areas and a thinner cortex in the frontal‐parietal region including the regions essential for creativity and executive processing. In particular, thinner cortex was evaluated in the math experts group in different regions: superior frontal right hemisphere (RH) and left hemisphere (LH), superior parietal RH, while larger surface area was evaluated in lingual (RH), superior frontal (RH and LH), and inferior parietal (LH) regions.^23^ In a review on neural correlates of mathematics in gifted adults, authors believed that the right hemisphere engagement during cognitive processing tasks might correlate with mathematical ability and the former electrophysiological findings are in line with the behavioral results.^24^ There is sufficient behavioral and cognitive literature about the individual differences in numeral comparison, which are considered as a strong predictor of arithmetic ability in school age children, but not enough in adults.^25^, ^26^ The present ERP information enables one to get a more fine‐grained picture than with behavioral outcomes alone.

Another important result of the current study is that the negative peak amplitude evoked by incorrect response was significantly greater at frontal, frontocentral, central, and centroparietal regions than correct response. This result extended the previous findings on number cognition ERP studies. For instance, in a research by Kong (2000), after 270 ms following the onset of the second stimulus (a number discrimination task, mismatching process of numbers), a negative component was seen with the highest peak amplitude at the midline central and occipital areas.^9^ In a similar study, false answers induced a negative component to the previous mental calculation in the time window between 202 to 340 millisecond.^8^ Zhou et al (2006) found a negative component (N240) at the same time window with a greater amplitude at the frontocentral sites in the non‐matched numbers than matched ones. Hsu and Szücs (2011) used number matching task and observed the AMN (Arithmetic mismatch negativity) at parietal electrodes. The AMN was larger in infrequent matching trials than frequent non‐matching trials. This ERP component showed the mismatch detection in reference to the strategic expectations violation processing.^10^ In previous studies, a negative component (N270), which appeared in the false response to the mental calculation, was stated. In the interpretation of the N270 production, the conflict of the mental information from the calculation in higher cortex and the upcoming information from the stimulus in the lower cortex was pinpointed.^8^, ^9^ In line with the literature, the AMN amplitude difference between two conditions might reflect a difference in activation between these two probe types. More negative amplitude may either considered as an additional need of attentional/processing, or alternately, characterize the mismatch detection between the representations of attended and expected stimuli. Hence, it might be better to clarify the AMN peak enhancement as a regular association of detecting stimulus mismatch, and not the index of expanded processing needs.^11^ This induction is in accordance with previous inferences stating that the N270 is a general correlation of conflict processing.

The mismatch negativity (MMN) component has been broadly used to research the pre‐attentive processing and storage of predictable basic features in stimulus (eg, spatial location, frequency). The MMN elicited if these predictabilities are violated. In the auditory cortices, this component has been preferred as an index of automated information processing. The information derived from this component appears to be in an implicit form and not clearly accessible to conscious processes. However, this unconscious process could influence the behavior of the individuals; for example, many studies showed MMN's different patterns in musicians and non‐musicians.^27^ In this regard, mental arithmetic is amodal process that does not rely just on auditory or visual in contrast to music. In general, the different AMN amplitude seen in high/low performers in this study might show the effect of arithmetic mastery on early cognitive process like strategic expectation. In summary, the results showed different brain activity for high/low math performance. In addition, mastery in abstract cognitive task like arithmetic affects the general and automatic process of strategic expectation.

## 5 CONCLUSIONS

Individual differences in math performance were illustrated by a negative potential as an ERP component while performing arithmetic verification task. The present results suggest that different math skills represent dissimilar degrees of a number‐processing specialization system that is probably involved in several complex interacting neural networks with a distinct topographic distribution. More generally, these data suggest that individual differences should be considered when studying the neurocognitive bases of mental arithmetic. In addition, the study showed the effect of math mastery on automatic expectation strategy. The ability to differentiate math mastery at neural level is probably beneficial in educational and clinical contexts where it is important to identify which process is responsible for the overt impairment.

## 6 LIMITATIONS

The limitations of this study highlight related subjects to be tended in future studies. First, while the findings of this study cannot be generalized to other tasks and experimental settings, it points an interesting aspect to consider in neuroeducational studies, which suggests that this aspect should be considered in these studies with different methods. Secondly, in ERP method artifacts confound the EEG data, thus artifact rejection impacts the electrophysiological expression of the evaluated processes.

## CONFLICT OF INTEREST

The authors of the present study declare that they have no conflict of interest.

## AUTHOR CONTRIBUTIONS

SH.T., AJ, TH, and MAN designed and conceptualized the study. SH.T. collected data. SH.T., AJ, TH, and MAN analyzed and interpreted data. SH.T. wrote the manuscript draft. AJ acted as corresponding author and all authors reviewed the manuscript.

## APPROVAL OF THE RESEARCH PROTOCOL BY AN INSTITUTIONAL REVIEWER BOARD

The study was approved by the local committee on human experimental research and was in accordance with the ethical standards of the 1964 Helsinki declaration and its later amendments or comparable ethical standards. The ethical committee of Tabriz University of Medical sciences approved this study (ID: IR.TBZMED.REC.1398.058).

## INFORMED CONSENT

Attendance was voluntary and all of the participants provided informed consent to complete the study protocol procedures.

## Data Availability

The data that support the findings of this study are available on request from the corresponding author. The data are not publicly available due to privacy and ethical restrictions because consent for all data directly associated with the results to be made available in a permanent, publicly accessible data archive, or repository was not obtained in the patient consent forms.
